# Evaluation of *Salmonella enterica* Serovar Typhimurium TTSS-2 Deficient *fur* Mutant as Safe Live-Attenuated Vaccine Candidate for Immunocompromised Mice

**DOI:** 10.1371/journal.pone.0052043

**Published:** 2012-12-17

**Authors:** Vikalp Vishwakarma, Niladri Bhusan Pati, Himanshu Singh Chandel, Sushree Sangeeta Sahoo, Bhaskar Saha, Mrutyunjay Suar

**Affiliations:** 1 School of Biotechnology, KIIT University, Bhubaneswar, Odisha, India; 2 National Centre for Cell Sciences, Ganeshkhind, Pune, India; International Center for Genetic Engineering and Biotechnology, India

## Abstract

*Salmonella enterica* serovar Typhimurium has been extensively exploited as live attenuated vaccines (LAV) which generally confers better protection than killed or subunit vaccines. However, many LAV are limited by their inherent ability to access systemic organs in many of the vaccinated hosts, especially those which are immunocompromised. We evaluated the efficacy of a live-attenuated SPI2-deficient (Δ*ssaV*) *S.* Typhimurium vaccine candidate (MT13) that additionally devoids the ferric uptake regulator (*fur*). We used specific pathogen free (SPF) streptomycin-pretreated mouse colitis model that included healthy C57BL/6 and immunocompromised *iNos* ^−/−^, *IL10^−/−^ and CD40L^−/−^* in the background of C57BL/6 mice to assess the efficacy of developed vaccine candidate. In our study, the *S*. Typhimurium MT13 strain was established as a safe vaccine candidate to be administered in immunocompromised mice as it was found to be systemically attenuated without conferring significant pathological signs and growth defect within the host. In bacterial challenge experiment, the MT13-vaccinated C57BL/6 mice were protected from subsequent wild-type *S*. Typhimurium infection by inducing proficient mucosal immunity. The MT13 strain elicited efficient O-antigen specific mucosal secretory IgA associated protective response which was comparable with its parental *ssaV* mutant. Vaccination with MT13 also showed proficient T-cell activation in host mice; which has direct relation with pathogen clearance from host tissues. Collectively, these data implicate the possible application of SPI-2 deficient *fur* mutant (MT13) as a novel live attenuated vaccine strain with adept immunogenicity and improved safety, even in immunocompromised hosts. Further, this vaccine candidate can be employed to express heterologous antigens targeted against several other diseases, especially related to enterocolitic pathogens.

## Introduction

*Salmonella enterica* is responsible for substantial economic losses worldwide due to the infections caused by its serovars and their subsequent management. Among all the reported serovars of *Salmonella*, Typhimurium and Enteritidis are the two major infectious agents involved in human gastroenteritis. These serovars signify zoonotic agents causing non-typhoidal salmonellosis (NTS) in broad host range. Vaccination represents a direct approach to lessen the rate of recurrence of *Salmonella* infections. With the progression in understanding the mechanistic basis of *Salmonella* infection, various genetically modified *Salmonella enterica* has emerged as a promising candidate for the development of attenuated live vaccine carrier strains [1]. Manifestation of disease from *Salmonella enterica* follows a complex process that involves numerous virulence factors, particularly two distinct type III secretion systems (T3SS) important for host-pathogen interactions [2]. The T3SS encoded by Salmonella Pathogenicity Island-1 (SPI1-T3SS) is required for successful invasion of non-phagocytic cells and is expressed by the extracellular bacteria. However, upon internalization, the intracellular *Salmonella* expresses proteins for the intracellular replication and systemic pathogenesis through another T3SS encoded by Salmonella Pathogenicity Island-2 (SPI2). It has been reported that more than 25 proteins encoded by SPI1-T3SS, plays their crucial role in cellular invasion. However, out of 25 kb long portion of the SPI2-T3SS involved in systemic spread of *Salmonella*, the two component system (*ssrAB*), structural component of TTSS (*ssa*), specific chaperons (*ssc*), putative substrate protein (*sse*) and other virulence proteins (*ssaV*, *ssaG*) are important for efficient functioning of SPI2-T3SS resulting subsequent systemic spread in the host tissues [2].

A successful live attenuated vaccine carrier strain should demonstrate efficient colonization to elicit mucosal immune response without any significant systemic infiltration. The basic techniques for the development of live attenuated vaccine carrier strains are well established [3], [4]. Nevertheless, very few live attenuated strains are approved for the human use and this is partially attributable to safety concerns arising from the use of vaccine strains in immunocompromised hosts [5], [6]. The safety risk associated with the vaccine strains can be addressed by such additional mutations in the genome of the bacterium which could restrict the *Salmonella* vaccine candidate from disseminating to host systemic tissues. Several *S*. Typhimurium mutants have been reported asserting potent protective efficacy for NTS in mouse colitis model [1], [7]–[11]. Inactivation of SPI1-T3SS renders the *Salmonella* dependent on alternative pathway to enter to non-phagocytic host cells; it might result in inefficient immune response. Alternatively, disrupting only the SPI2-T3SS would avert the access of mutant strain to systemic organs of the host, thus making the strain attenuated. However, the SPI-2 deleted *Salmonella* has also been reported with virulent properties [12]. C57BL/6 mice vaccinated with the *S*. Typhimurium *ssaV* mutant, showed O-antigen specific responses and were protected from subsequent *S*. Typhimurium challenge infections [13], [14]. However, in this study, introduction of *S.* Typhimurium SPI2-T3SS mutant to immune-compromised mice (IL10^−/−^, CD40L^−/−^, *iNos* ^−/−^) indicated that the mutant deficient in T3SS-II is not safe enough and caused lethal infections. We have tried to address the possibility to identify additional mutation that could further attenuate the *ssaV* mutant and confer protection in mouse colitis model.

Systemic spread of *S*. Typhimurium *ssaV* mutant, in host system, implicates some metabolic proteins favoring the survival of the bacterium. Immense pH variation is one of the many physiological challenges that *Salmonella* encounters while being in stomach, movement towards gut and invading through the intestinal epithelia or when captured in *Salmonella* containing vacuoles (SCV) in the host macrophages. One such metabolic protein responsible for acid tolerance and iron acquisition is encoded by *fur* (ferric uptake regulator) in *S*. Typhimurium [15]. Iron functions as a cofactor in several metabolism-associated enzymes. Alteration in expression of SPI1 proteins by direct interaction with the *hilD* operator and *hilA* regulator is another activity from *fur* [16], [17]. Fur is a well-characterized transcriptional repressor that regulates gene expression in response to iron and maintains iron homeostasis in many bacteria [18]. It has been shown that the Fur is involved in regulating genes related to antioxidant enzymes like iron superoxide dismutase (FeSOD) and manganese superoxide dismutases (MnSOD), histone-like protein H-NS and other huge number of metabolic proteins as novel targets of Fur in *Salmonella* Typhimurium [19]. Protection of DNA against oxidative stress is another crucial role of *fur* by direct upregulation of Dps (DNA binding protein in stationary phase) [20]. Taken together, existing literature proposes for a diverse role of *fur* in combating host environment towards bacterial survival, alterations in expression of various genes associated with host metabolic drifts and modulation of effector proteins secreted through SPI1-T3SS.

In conclusion, present study addresses the incorporation of additional mutation in *fur* gene in SPI2 deficient *Salmonella* which focuses on the design of effective live attenuated vaccine that would be safe for vaccination to immunocompromised mice and while being immunogenic enough to confer protection against *Salmonella* infection. Further, such attenuated vaccine strains can be used as safe vaccine carrier strain and to design a polyvalent vaccine with reduced risk of infections in immunocompromised hosts.

## Materials and Methods

### Bacterial Strains and Growth Condition

Bacterial strains (Table 1) were grown for 12 h at 37°C in Luria-Bertani medium (LB) supplemented with 0.3 M NaCl, diluted 1∶20 in fresh LB medium and subcultured for another 4 h under mild aeration until an optical density of 0.6 was obtained. The bacteria were washed in ice-cold phosphate buffered saline (PBS) and 5×10^7^ CFU were suspended in 50 µl cold PBS for use in the *in vivo* experiments. Prior to the start of the *in vivo* experiments, all strains were tested for growth attenuation for 16 h in 10 ml of culture medium at 37°C with 150 rpm under aerated conditions.

**Table 1:** Bacterial strains, plasmids and primers used in study.

Strains	Genetic information	Background	References
SB300	*Salmonella* Typhimurium; *Sm^r^*	Wild-type	[24]
M1525	*Salmonella* Enteritidis 125109 wild type; *Sm^r^*	Wild-type	[30]
MT11	*S.* Typhimurium *fur::aphT*; *Sm^r^, Km^r^*	SB300	This study
MT12	*S.* Typhimurium Δ*ssaV*; *Sm^r^*	SB300	This study
MT13	*S.* Typhimurium *fur::aphT,* Δ*ssaV*; *Sm^r^, Km^r^*	MT12	This study
**Plasmids**	**Relevant genotype (S) and/or phenotype (S)**	**Resistance**	**References**
pM973	*bla* P*ssaH* gfpmut2 plasmid with *ori*pMB1	Amp^r^	[24]
pKD46	Red recombinase expression plasmid; P*_araB_;* oriR101	Amp^r^	[20]
pKD4	Template plasmid; FRT-*aphT*-FRT	Km^r^	[20]
pCP20	FLP recombinase expression plasmid	Cm^r^, Amp^r^	[20]
**Primers**	**Sequence (5′ to 3′)**		
Fw-*fur*	GATATAAAAAAGCCAACCGGGCGGTTGGCTCTTCGAAAGATTTACACTGTGTAGGCTGGAGCTGCTT		
Rw-*fur*	CACTTCTCTAATGAAGTGAATCGTTTAGCAACAGGACAGATTCCGCATATGAATATCCTCCTTAGT		
Conf-*fur*	GCCAATATCAAGATCCTGTGCG		
Fw-*ssaV*	TCATCGACAAATAAAATTTCTGGAGTCGCAATGCGTTCATGGTTATGTGTAGGCTGGAGCTGCTT		
Rw-*ssaV*	ATTTCAGCCTCAGACGTTGCATCAATTCATTCTTCATTGTCCGCCATATGAATATCCTCCTTAGT		
Conf-*ssaV*	GCAATGAGTTGTTCTCCACC		

### Chromosomal Gene Disruption

Deletions of *Salmonella* Typhimurium genes *fur* (MT11; *fur::aphT*) and *ssaV* (MT12; Δ*ssaV*) were materialised using standard lambda-red recombinase system with pKD46, pKD4 template plasmid and pCP20 flip-recombinase plasmid [21], [22] using the primers listed in Table 1. The phage lysate of the developed mutants were prepared, and the stable double mutant strain MT13 (*fur::aphT*, Δ*ssaV*) was obtained by transducing the *fur::aphT* mutation into the recipient MT12 (Δ*ssaV*) strain. All the mutations were confirmed by PCR using the *fur*- and *ssaV*-specific confirmatory primers (Table 1).

### Ethical Statement

All the animal experiments were performed in strict accordance with guidelines laid by the Institutional Animal Ethics Committee (IAEC) of National Center for Cell Sciences (NCCS) Pune, India. This study was approved by the IAEC of NCCS; Permit Number: 7/1999/CPCSEA-09/03/1999. All efforts were made to minimize suffering of animals during experimentation.

### Mice Infection Experiment for Assessment of Strain Attenuation

The infection experiments were performed in individually ventilated cages as described previously [23]. All mice were specific pathogen free (SPF). C57BL/6, *iNos* ^−/−^ (B6.129P2-*Nos2tm1Lau*/J), *IL10^−/−^* (B6.129P2-IL10^tm1cgn^/J) *and CD40L^−/−^* (B6.129S2-Cd40lg^tm1Imx^/J) mice from Jackson Laboratories (Bar Harbor, ME) were bred in the C57BL/6 background at the animal facility of NCCS, Pune. Mice were pretreated intragastrically with 50 mg of streptomycin before infecting with *Salmonella*. After 24 h, mice were infected with 5×10^7^ CFU (oral gavage) of the corresponding bacterial strain (i.e. MT12, MT13 and SB300). The bacterial loads in the cecum, mesenteric lymph nodes (MLNs), liver and spleen were determined by plating the respective tissue homogenates on MacConkey agar plates supplemented with appropriate antibiotics (streptomycin; 50 µg/ml, kanamycin; 50 µg/ml, ampicillin; 20 µg/ml, chloramphenicol; 20 µg/ml).

### Vaccination and Challenge Experiment

Vaccination experiments were carried out in streptomycin pre-treated C57BL/6 mice as established previously [23]. Briefly, three mice groups (n = 8) were vaccinated with MT12 and MT13 *S.* Typhimurium strains; the PBS treated mice group served as control population. The fecal samples from each mouse were collected at various time points and their suitably diluted suspensions were spread on MacConkey agar for enumerating bacterial shedding. Also, the bacterial loads in the cecum content and other designated organs like mLN, spleen and liver were determined at day 30 post vaccination (p.v.) by sacrificing mice (n = 4) from each group. For statistical analysis, samples with no bacterial count were adjusted to the minimal detectable levels. Remaining mice (n = 4) from each vaccinated group were treated with ampicillin (25 mg) to clear the residual *Salmonella* or other microbial flora from mice gut; challenged after 24 h by infecting them with 200 cfu of pM973-harbouring *Salmonella* Typhimurium SB300 wild-type strain. The plasmid pM973 was used to maintain the ampicillin resistance in wild-type SB300 strain during the course of challenge experiment [24]. At day 3 post challenge (p.c.), the colonization efficiency of challenge strain SB300 at various host sites was assessed. The tissues were also collected in an Optimum Cutting Temperature compound (OCT, Sakura Finetek USA Inc.), snap frozen in liquid nitrogen and stored at −80°C for cryosectioning. Cecal inflammation of the infected tissue was scored as described further.

### Histopathological Evaluation

Segments of the ileum, cecum, and colon were fixed and embedded in O.C.T. (Sakura Finetek Inc., USA), snap-frozen in liquid nitrogen, and stored at −80°C. The cryo-embedded cecal tissue sections (5 µm) were sectioned at −30°C and collected on glass slides and stained with hematoxylin and eosin (H&E) after drying for at least 2 h at room temperature. The stained histopathological sections were evaluated on the basis of a previously described scoring system for the quantitative analysis of cecal inflammation [23]–[25]. The H&E-stained sections (5 µm) were scored independently on the basis of pathological changes that include sub-mucosal edema (0–3), polymorphonuclear leukocyte infiltration (0–4), loss of goblet cells (0–3) and epithelial ulceration (0–3) with a summation score that range between 0–13, reflecting the degree of inflammation. The independent pathoscores of each tissue sample were averaged and combined. The combined pathological scores ranged from 0 to 13 arbitrary units covering the inflammation levels that included intact intestine without any sign of inflammation (pathoscore 0); minimal sign of inflammation (pathoscore 1–2), which is commonly found in the ceca of specific pathogen-free mice and generally not considered as a pathological feature; slight inflammation as a minimal sign of tissue pathology (pathoscore 3–4); moderate inflammation (pathoscore 5–8); and significant inflammation (pathoscore 9–13) [26].

### FACS Analysis for T-cell Population

FACS analysis of different T-cell populations was performed by collecting the mesenteric lymph node (mLN) from the immunized mice at day 4 and day 30 p.c. The mLN were collected in 500 µl of RPMI medium (Lonza, USA) supplemented with 10% fetal calf serum (FCS; Lonza, USA). The mLN were homogenized and centrifuged at 140×g for 8 min at 4°C. The cellular fractions were collected and resuspended in 1 ml RPMI-FCS medium. Around 10^6^ cells were resuspended in 50 µl of FACS buffer and incubated with different T-cell specific antibodies (PE-anti mouse αCD3, BD Pharmingen; PB- anti mouse αCD4, BD Pharmingen; FITC-anti mouse αCD8, BD Pharmingen) for 1 h and 30 min at room temperature. The cells were washed and fixed with 1% paraformaldehyde (in PBS) for 15 min at 4°C. Finally, the cells were washed and the cell surface staining was assessed by FACS Canto (BD Biosciences) and analyzed by FACS Diva software (BD Biosciences).

### Assessment of Serum and Gut Antibody Response

As a measure of mucosal immune response, serum IgG and luminal secretory IgA responses were qualitatively assessed by Western blotting and FACS analysis as described previously [13], [14]. Serum and gut washes from MT12 and MT13 immunized mice groups were collected at 30 days p.v. and stored after centrifuging them at 9′100×g for 10 min in a cooling centrifuge at 4°C. Bacterial lysates of the overnight-grown *Salmonella* Enteritidis P125109 wild-type strain (M1525), *Salmonella* Typhimurium wild-type strain (SB300), *fur* deficient (MT11), *ssaV* deficient (MT12) and *fur, ssaV* double deficient (MT113) mutant strains were separated on polyacrylamide gels; transferred to nitro-cellulose membrane. The membrane was developed with suitably diluted samples of serum or gut washes followed by incubation with secondary antibodies (Santa Cruz Biotechnology, USA) for α-mouse IgA (for gut wash) and α-mouse IgG (for serum) after intermittent membrane washing.

For FACS analysis of serum IgG and luminal IgA responses, single colony of *Salmonella* Typhimurium (SB300) was grown overnight in static LB broth at 37°C. Bacterial cells, obtained from centrifuging 1 ml of overnight grown culture, were washed twice with sterile PBS supplemented with 1% BSA and 0.05% sodium azide; and finally suspended in PBS with no additives to have bacterial density 10^7^ cfu/ml. For assessing serum IgG response, the diluted mice serum samples were inactivated at 60°C for 30 min and clear supernatant was diluted to 1∶20, 1∶60 and 1∶120. Similarly, for secreted IgA, the clear supernatants of luminal contents were kept as undiluted, 1∶3 and 1∶9 dilutions in PBS. Further, a 25 µl of diluted serum samples and the gut wash were mixed separately with 25 µl bacterial suspension of *Salmonella* Typhimurium (SB300) in PBS and incubated for 1 h at 4°C. The bacterial cells were washed twice with PBS, reacted with FITC-conjugated monoclonal anti-mouse IgG and IgA antibodies (Abcam, USA) for serum and gut-wash samples respectively, and incubated for 1 h at 4°C. Finally, cells were washed once with PBS (1% BSA, 0.05% sodium azide) and resuspended in PBS (2% PFA). Acquisition was performed using FACS Canto analyser (BD Biosciences, USA).

### Statistical Analysis

Statistical analyses were performed using t-test and other suitable statistical analysis parameters (Prism 5; GraphPad Software, La Jolla California, USA) when needed. Probability with P<0.05 was considered statistically significant.

## Results

### *S.* Typhimurium SPI2-deficient *fur* Mutant was Attenuated in Immunocompromised Mice

The SPI2-TTSS deficient *Salmonella* Typhimurium strain has already been established as safe vaccine [13], [14]. Though it gives protective mucosal immunity in C57BL/6 mice but it causes lethal infections in various isogenic immunocompromised mice in our experiments. Additional mutations attenuating the modified vaccine strain in immunocompromised mice while retaining its immunogenic properties would be a great benefit to circumvent the problem.

**Figure 1:**
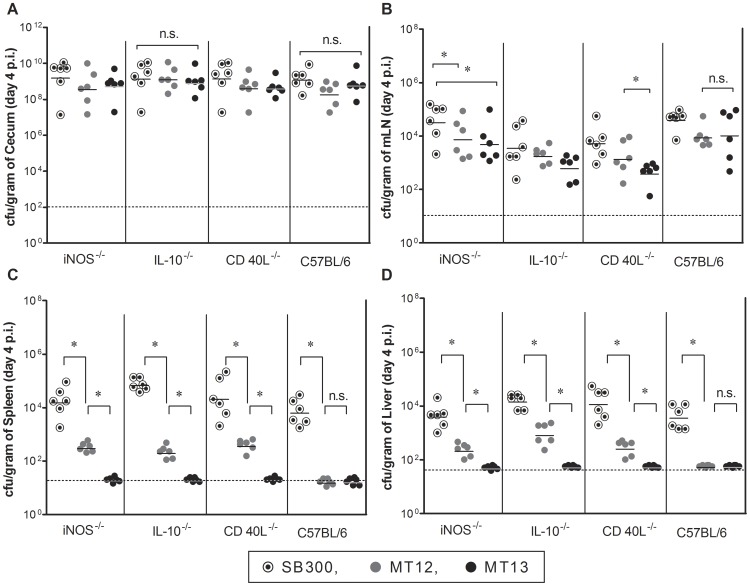
Attenuation status of MT12 and MT13 *S*. Typhimurium strains in immunocompromised mice. To identify the attenuation properties of developed *Salmonella* strains, groups of various immunocompromised mice (*iNos^−/−^, IL10^−/−^, CD40L^−/−^* in C57BL/6 background along with wild-type mice as control group) were infected with 5×10^7^ cfu of different strains of *Salmonella* Typhimurium (wild-type: SB300; *ssaV* mutant: MT12; *ssaV* and *fur* double mutant: MT13) by gavage. Mice were sacrificed at 4 days p.i. and bacterial colonization was assessed. Graph represents colonization of *S.* Typhimurium strains at different host sites; **A:** cecum, **B:** mesenteric lymph nodes, **C:** spleen and **D:** liver. Broken lines in the graphs shows minimum detection limit. n.s.: not significant, *: statistically significant (P<0.05, t-test).

Attenuation of *S.* Typhimurium strains was analysed in C57BL/6, isogenic immunocompromised *iNos^−/−^*, *IL10^−/−^* and *CD40^−/−^* mice at day 4 p.i. Animals were infected with *Salmonella* Typhimurium SB300 (wild-type strain), MT12 and MT13 strains. We monitored the intestinal colonization and systemic spread of introduced bacterial strains in various host tissues like mesenteric lymph nodes, spleen and liver. All the strains colonised efficiently in the gut of C57BL/6 as well as other immunocompromised mice (Fig. 1A). The bacterial densities of MT12 and MT13 were found to be statistically significant with SB300 counts in mLN of all the mice groups (Fig. 1B). However, the SPI2-deficient *fur* mutant was marginally attenuated in mLN of other immunocompromised mice (Fig. 1B). SPI2-deficient MT12 strain was highly attenuated in spleen and liver of infected C57BL/6 mice group but it was showing significant counts in the systemic sites (liver and spleen) of isogenic immunocompromised mice (Fig. 1C, 1D). In contrast, the MT13 was strictly attenuated in C57BL/6 as well as other immunocompromised mice groups (Fig. 1C, 1D). Collectively, these data proposes the SPI2-deficient *fur* mutant (MT13) as a safer vaccine candidate which can be used in different immunocompromised mice intruding the problems of infections by vaccine strain itself.

### The *fur* Mutation in SPI2-deficient Strain Doesn’t Compromise the Intestinal Colonisation Efficacy

**Figure 2:**
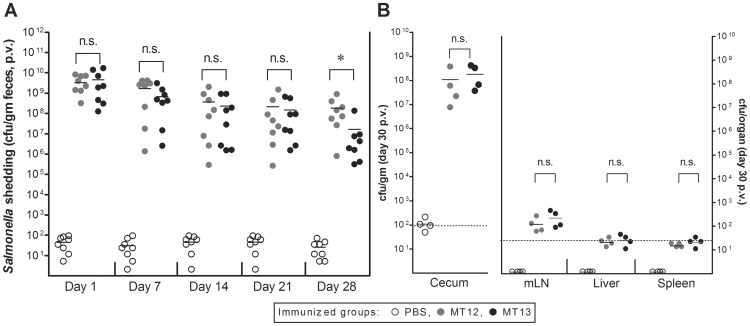
Colonisation of vaccine candidate and its systemic access. Vaccination-challenge experiment to analyze the immunogenic potential of *S.* Typhimurium M12 and MT13 strain. Groups of C57BL/6 mice were vaccinated with 5×10^7^ cfu of *Salmonella* Typhimurium MT12 (*ssaV* mutant; closed grey circles; n = 8) and MT13 (*ssaV* and *fur* double mutant; closed black circles; n = 8) by gavage. One group of mice was treated with PBS (open circles; n = 8) to serve as control. Mice groups were kept under observation for next 30 days. **A:** Fecal shedding of *Salmonella* strains as analyzed by plating at various time-points in days. Four mice from each vaccinated group were sacrificed at day 30 post-vaccination (p.v.) to assess the bacterial population at different host sites. **B:** Enumeration of bacterial loads at various host sites. Broken lines in the graphs shows minimum detection limit. n.s.: not significant, *: statistically significant (P<0.05, t-test).

To have an effective and prolonged mucosal immune response is the ultimate prerequisite of an efficient mucosal vaccine candidate. To meet the criteria, the vaccine candidate must colonise efficiently in host gut for a prolonged time to elicit better mucosal immunity. Keeping this in view, groups of C57BL/6 mice were vaccinated with *S.* Typhimurium MT12 and MT13 strains. The PBS treated mice served as negative control group and the bacterial counts from PBS treated mice group were set at minimum detection limits to facilitate proper statistical analysis. We monitored the fecal shedding of introduced *Salmonella* strains at various time points during the course of vaccination. Both of the strain attained cecal densities of ∼10^9^ cfu/g as early as day 1 p.v. and the counts were found to decline at equivalent rates afterwards (compare day 1, 7, 14, 21). The MT12 and MT13 were shed at comparable rate until day 21 p.v., showing their comparable colonization efficiency in the gut of host mice. However, at day 28 p.v., the fecal shedding of strain MT13 was found to be slightly reduced (P<0.05) in comparison with MT12 (Fig. 2A). To confirm the findings, cecal bacterial loads were analysed at day 30 p.v. from immunized mice groups (n = 4; each group). We observed comparable cecal densities of MT12 and MT13. Also, both the strains were statistically comparable at different systemic sites of vaccinated C57BL/6 mice groups (Fig. 2B). The negative control mice group did not show any signs of *Salmonella* infiltrations; however, the counts were kept at minimum detection levels to facilitate appropriate statistical analysis. Collectively, efficient colonisation in gut associated mucosa and the draining lymph node by SPI2-deficiet fur mutant (MT13) suggested that the vaccine strain retained its immunogenic potential in C57BL/6 mice.

### MT13 Vaccination Protected C57BL/6 Mice from Subsequent Wild-type *S.* Typhimurium Challenge

**Figure 3:**
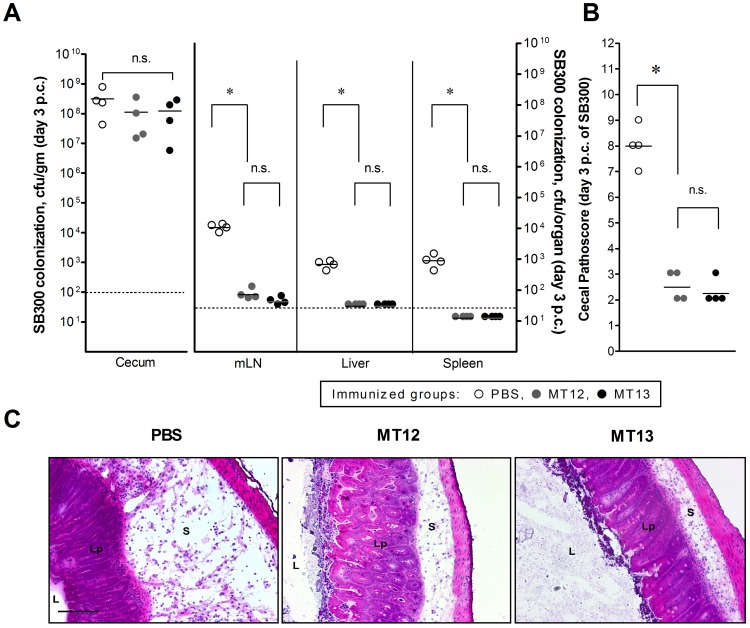
*S.* Typhimurium challenge experiment: an assessment of vaccination efficacy. Vaccinated C57BL/6 mice groups (PBS: n = 4, MT12: n = 4 and MT13: n = 4) were ampicillin-treated (25 mg by gavage), challenged with wild-type SB300 (amp^r^, sm^r^) and euthanized at day 3 post-challenge (p.c.) i.e. day 34 post-vaccination. **A:** Colonization at various host sites after SB300 challenge. **B:** Enumeration of cecal pathology in terms of 13 point pathoscore scale was determined as described above (see material and methods). Broken lines in the graphs shows minimum detection limit. n.s.: not significant, *: statistically significant (P<0.05, t-test). **C:** Representative HE-stained cecum sections obtained from SB300-challenged mice groups immunized with PBS, MT12, MT13. Bar, 200 µm. S, submucosal edema; Lp, lamina propria; L, lumen.

An efficient vaccine candidate should primarily be able of protecting vaccinated host from the lethal infections of isogenic wild-type strains. To assess the vaccination potential of MT13, the remaining mice (n = 4) from each vaccinated group were treated with ampicillin (20 mg) to remove any residual *Salmonella* and re-grown gut flora. Mice were challenged, after 24 h, with SB300 (wild-type *Salmonella* Typhimurium) strain by gavage. Mice were sacrificed at day 3 p.c. and the bacterial densities at various host sites and cecal pathology was assessed. The challenge strain (SB300) colonised efficiently in the gut of each vaccinated mice and the bacterial counts were comparable even with the PBS treated control vaccinated mice group (P>0.05). On assessing systemic sites, we observed that SB300 had enhanced access to mLN, spleen and liver of PBS treated control group of mice as compared to the groups vaccinated with MT12 and MT13 (Fig. 3A). It was observed that PBS treated mice could not develop any protective immunity to combat *Salmonella* infections. However, the SB300 could not colonize in mLN, spleen and liver of MT12 and MT13 vaccinated mice group indicating the development of effective immunity in such immunized mice (Fig. 3A). Cecal pathoscores of MT12 and MT13 vaccinated mice were also found to be in-line with above observations (Fig. 3B) and was also supported by the HE-stained representative cecal tissue sections (Fig. 3C). Taken together, it was shown that the vaccination by SPI2-TTSS deficient *fur* mutant had the ability to protect C57BL/6 mice from subsequent lethal infections of wild-type *Salmonella* Typhimurium.

### SPI-2 Deficient *fur* Mutant has an Ability to Induce Efficient T-cell Proliferation

**Figure 4:**
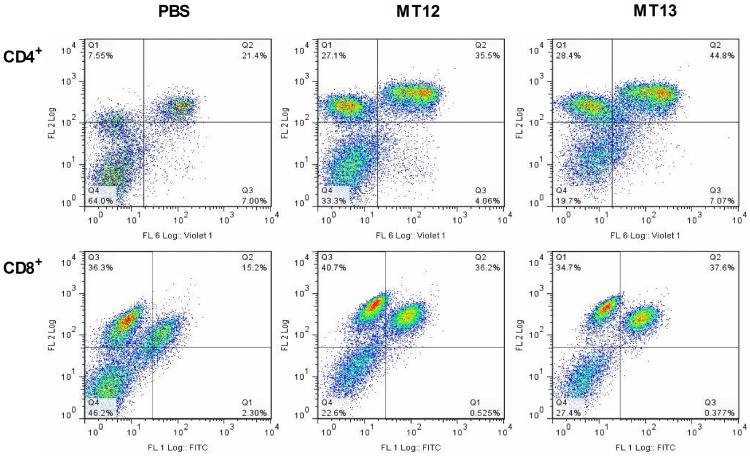
Evaluation of T-cell immune responses from vaccinated mice groups. Tissue homogenates of mesenteric lymph nodes (mLN), collected from mice groups vaccinated with MT12, MT13 and PBS at day 30 p.v., were FACS analyzed for T-cell response. Representative FACS graphs depicting CD4^+^ (pacific blue labelled) and CD8^+^ (FITC labelled) cell populations (second quadrant in each graph) in various immunized mice groups. Serum and mucosal antibody response from immunized mice was analyzed; serum and gut wash were collected at day 30 p.v.; bacterial lysate of overnight cultures of SB300 (wild-type *S.*Typhimurium), M1525 (wild-type *S.* Enteritidis P125109), MT11 (S.Tm *fur* mutant), MT12 (S.Tm *ssaV* mutant) and MT13 (S.Tm *ssaV* and *fur* double mutant) were developed with serum or gut wash from immunized mice.

An ideal live attenuated mucosal vaccine strain should have the capacity to induce immunogenicity by accessing host gut associated secondary lymphoid tissues. To analyse this attribute, the effect of SPI-2 deficient (MT12) and additionally *fur* knocked-down double mutant (MT13) on proliferation of T- cell in the mesenteric lymph node was assessed. The change in T-helper cells in the mLN between day 4 (data not shown) and day 30 p.v. was analyzed. A marked increase in the CD4^+^ and CD8^+^ T-cell population was observed in MT12 and MT13 vaccinated mice groups as compared to PBS treated samples at day 30 p.v. (Fig. 4). Overall, results indicated comparable efficiency of SPI-2 deficient *fur* mutant to induce T-cell response in the MT13-vaccinated C57BL/6 mice.

### MT13-vaccinated Mice Showed Efficient Luminal and Serum Antibody Responses

**Figure 5:**
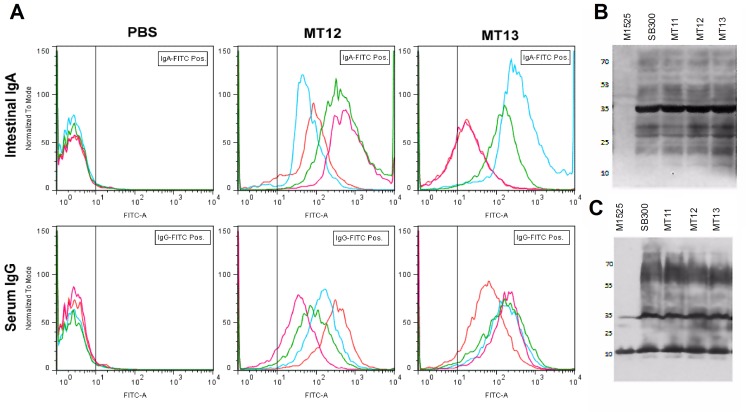
Evaluation of intestinal and serum immune responses from vaccinated mice groups. **A:** Assessment of antibody response (serum IgG and intestinal IgA) by bacterial FACS. Gut-wash/serum samples from the mice immunized with PBS (n = 4), MT12 (n = 4) or MT13 (n = 4) were serially diluted and analyzed by FACS. Representative FACS graphs for IgG response from diluted (1∶120) serum sample and intestinal IgA response from diluted (1∶9) sample from gut-wash are shown. Each plotted line in a panel (PBS/MT12/MT13) indicates data from individual mice of a group. **B:** Representative western blots of mucosal secretory IgA response and **C:** serum IgG response as observed on final treatment with α-mouse IgA (for gut wash) and α-mouse IgG (for serum).

It has been previously established that immune-protection by SPI2 deficient *Salmonella* strain basically develops through luminal secreted IgA (sIgA) specific to *Salmonella* O-antigens. This immune status can also be found as serum IgG response [14]. To validate the immunogenic potency of SPI-2 deficient *fur* mutant (MT13), we analyzed the serum and luminal antibody response of MT13-vaccinated mice against *Salmonella* infection by FACS analysis and Western blotting. For FACS analysis, serum and gut wash samples from mice were reacted with *S*. Typhimurium (SB300) (see Materials and Methods). We observed admirable IgG and IgA antibody response against SB300 from the serum and gut-wash samples obtained from mice immunized with MT12 and MT13; however, neither IgG nor IgA response (specific to *S*. Typhimurium; SB300) was observed in case of mock (PBS) immunised mice (Fig. 5A). MT13 and MT12 immunized mice showed comparable antibody response as seen after FACS analysis of highest diluted (1∶120) serum and (1∶9) gut-wash samples. We observed similar pattern of response with other dilutions (serum; 1∶20, 1∶60 and gut-wash; undiluted, 1∶3) of the samples (data not shown). Above observations were also validated by Western blotting experiment where bacterial crude lysate of overnight grown *Salmonella* Typhimurium (SB300, MT11, MT12 and MT13 test samples) and *Salmonella* Enteritidis (M1525; wild-type as negative control) were run on polyacrylamide gels and reacted with collected sample of mice serum or gut wash. The blots were developed with suitable secondary antibodies specific to IgG or IgA. In both of the blots, the gut wash and serum samples reflected efficient luminal (Fig. 5B) and serum (Fig. 5C) antibody response against all the subjected variants of *Salmonella* Typhimurium. However, these host antibody samples could not show any response against *S*. Enteritidis specific O-antigens. Appearance of IgA and IgM specific reactions in Western blots were strongly suggestive of *S.* Typhimurium O-antigen specific luminal and serum antibody response after vaccination with SPI-2 deficient *fur* mutant.

## Discussion

Non-typhoidal Salmonellosis (NTS) is endemic to rural and urban parts of many developing countries. Certain prevalent diseases and their symptoms increases susceptibility to NTS infections [27]–[29]. Young children and human immunodeficiency virus (HIV) infected people have a high risk to develop bacteraemia upon NTS infection [28], [29]. For the treatment of childhood sepsis the World Health Organization (WHO) advises treatment with antimicrobial agents. However, many NTS strains are already resistant to common recommended antibiotics [30]. Till date, there are no protective vaccines available for NTS infections. Hence, designing of efficient vaccines that could protect animals and humans, especially immune-compromised hosts, would be of great benefit to combat *Salmonella* infections. The design and construction of a number of rationally attenuated *Salmonella* vaccine strains having potential to protect against typhoid infection have already been explained by use of *S. *Typhimurium murine infection models [31], [32]. The use of live attenuated strains is more promising, since it induces a relatively stronger immune response than killed organisms [33]. Many of the contemporary strategies for development of *Salmonella* vaccines and vaccination methodologies have been explained [7], [34], [35]. Several rationally attenuated live vaccines, such as strains deficient in stress responses (*htrA*), auxotrophic mutants (*aroA*, *aroCD*, *pur A*), or adenylate cyclase mutants (*cya, crp*) have been investigated as vaccine candidates [36]. Other strains having mutations in virulence genes or their regulators were also explored for their effectiveness as vaccines including *Salmonella* type-III secretion system-2 (TTSS-2) protein SsaV [37], [38]. The *ssaV* deleted *S*. Choleraesuis mutant strain was claimed superior to commercially available live vaccines by being safe and providing effective immune response against subsequent challenge [39]. However, in few of the studies, the infection by *ssaV* mutant strain caused substantial increase in water loss through feces of infected mice without showing any pathological changes in colitis [38]. For this reason, many *Salmonella* mutants failed either to prevent systemic dissemination of the bacteria or to induce effective immune response. These outlooks signify a setback in vaccine development and our study addresses the possibility of designing further safer vaccine candidates without compromising their immunogenicity.

The ferric uptake regulator (Fur) is the primary iron regulatory protein in *Salmonella* and *Escherichia coli*, and homologs of Fur have been found in other bacterial strains too. Fur modulates SPI-1 expression by controlling its HilA and HilD regulators. Also, the Fur regulon includes genes involved in regulation of various cellular functions comprising iron acquisition, acid-tolerance, sugar metabolism, defence against oxygen radicals, and genes encoding bacterial toxins [16], [40], [41]. Fur has also been reported for activation of virulence gene expression in pathogenic bacteria and its possible related mechanisms have been explained [17], [41]–[43]. The promoter of *fur* has also been exploited in developing vaccines with regulated delayed attenuation [44]. Above properties suitably explains the selection of additional *fur* mutation in TTSS-2 deficient *Salmonella* Typhimurium to develop a safer vaccine candidate.

A pathogen needs to take-up iron from the host during infection as it is obligatory for bacterial survival and multiplication within the host. However, the level of freely available iron is kept limited by host high-affinity iron binding proteins. To conquer this situation, bacterial pathogens have developed iron uptake systems, typically tightly controlled by the ferric uptake regulator (Fur) protein [45]. In our findings, we demonstrated the fine-attenuation of SPI-2 deficient *fur* mutant (MT13) in various immunocompromised mice groups as compared to its parental mutant strain MT12. It was interesting to note that MT13 was attenuated not only in the *iNos^−/−^* mice, but also in animals deficient for IL10 based inflammatory cytokines response and the CD40-Ligand. Thus, the safety of MT13 was enhanced in many immuno-deficient hosts. Hence, the conferred attenuation by *fur* deletion in SPI-2 deficient strain implicates the safety of MT13 as an improved live attenuated vaccine strain in immunocompromised hosts. The attenuation of MT13 is possibly attributed to inactivation of *fur*, which is reported to limit the bacterial growth in low iron conditions prevalent in systemic organs.

In the context of immunity against persistent infections, the balance between immune activation and immune suppression is crucial for fine-tuning host defence and to limit host-injuries. The T-cells ability to modulate the balance between bacterial clearance and proliferation has also been established [46] and the in vivo activation of T-cells during *Salmonella* infections have already been reported [47], [48]. The CD4^+^ T-cells mediate the clearance of the bacteria from the tissues. It is likely that the T-cell response confers protection via activation of the mononuclear cells where *Salmonella* resides. On the other hand, the CD8^+^ T-cells differentiate into cytotoxic T-lymphocytes, which may also play a role in protection by liberating intracellular *S*. Typhimurium from infected macrophages [49]. Thus, the activation of Th1 cells is required not only for the defence against primary infection with *Salmonella* but also for the vaccine-induced resolution of infection [50]. In our findings, we have shown that CD4+ and CD8+ T-cells proliferation increases after 30 days vaccination of host mice. The increase in T-cells count was in-line with above discussion and it could be considered partially, apart from other well studied mechanisms, to confer partial protection against subsequent *Salmonella* challenges.

At the level of mucosal immune response; secretory IgA (sIgA), the predominant antibody class in external secretions, plays a key role in immune protection. Several mechanisms of sIgA function in the intestinal lumen have been described. Studies have revealed that sIgA interferes with pathogen adhesion and invasion by binding to epitopes present on the surface of pathogens or toxins impairing attachment to epithelial cell receptors. This mechanism has been shown for cholera toxin [51], capsid protein of reovirus [52] and other infectious agents. In our *in vivo* findings, O-antigen specific sIgA response seemed to prevent the entry of *S. *Typhimurium to systemic sites of immunized mice. Access of *S. *Typhimurium to spleen and liver was significantly reduced in SPI2-deficient (MT12) and SPI2-deficient *fur* mutant (MT13) vaccination. In our study, the samples collected from the immunized and challenged C57BL/6 mice responded to *S*. Typhimuirum specific serum IgG and luminal sIgA antibodies against the O-antigen. It has already been established that the protection against *Salmonella* secondary infection depends on O-antigen-specific sIgA and required both B- and T-cells activation [14], [53]. However, detailed understanding of the functions of sIgA in mucosal immunity would be necessary for rational design of efficient mucosal vaccines.

In conclusion, the live-attenuated *Salmonella* Typhimurium vaccine strain MT13, which comprised deletion mutation of ferric-uptake regulator (*fur*) in SPI-2 non-functional strain, was found to be attenuated in various immunocompromised mice hosts. The same was found to be competent enough to induce efficient serum and mucosal immune responses to confer protections against wild-type *Salmonella* Typhimurium infections. Overall, the present study addresses the possibility for development of new live attenuated vaccines with improved safety and immunogenicity, particularly targeting the immunocompromised hosts. It would be interesting to carry a comparative analysis of the mechanisms backing attenuation status of MT13 and MT12 in experimental hosts of different genetic background. Also, the in-depth work on protective mucosal immunity by MT13 vaccination could possibly explore new immunological niche to explain protective immunity mounted by the strain. Further, the expression of heterologous antigens from various pathogenic organisms, through the vaccine strain MT13 would be in prime focus for future application of this strain as polyvalent vaccine to combat various diseases, especially those which are caused by enterocolitic pathogens.
